# Surgical treatment of Neer type II and type V lateral clavicular fractures: comparison of hook plate versus superior plate with lateral extension: a retrospective cohort study

**DOI:** 10.1007/s00590-019-02411-9

**Published:** 2019-03-07

**Authors:** Yassine Ochen, Herman Frima, R. Marijn Houwert, Marilyn Heng, Mark van Heijl, Egbert J. M. M. Verleisdonk, Detlef van der Velde

**Affiliations:** 1 Department of Surgery, University Medical Center Utrecht, PO Box 85500, 3508 GA Utrecht, The Netherlands; 2 Department of Surgery, St. Antonius Hospital, Nieuwegein, The Netherlands; 3 Department of Orthopedic Surgery, Harvard Medical School Orthopedic Trauma Initiative, Massachusetts General Hospital, Boston, USA; 4 Department of Surgery, Kantonsspital Graubünden, Chur, Switzerland; 5 Department of Surgery, Diakonessenhuis Hospital, Utrecht, The Netherlands; 6 Department of Surgery, Academic Medical Center, Amsterdam, The Netherlands

**Keywords:** Lateral clavicle fracture, Unstable, Plate fixation, Clavicle hook plate, Superior clavicle plate with lateral extension

## Abstract

**Purpose:**

Different fixation methods are used for treatment of unstable lateral clavicle fractures (LCF). Definitive consensus and guidelines for the surgical fixation of LCF have not been established. The aim of this study was to compare patient-reported functional outcome after open reduction and internal fixation with the clavicle hook plate (CHP) and the superior clavicle plate with lateral extension (SCPLE).

**Methods:**

A dual-center retrospective cohort study was performed. All patients operatively treated for unstable Neer type II and type V LCF between 2011 and 2016, with the CHP (*n* = 23) or SCPLE (*n* = 53), were eligible for inclusion. The primary outcome was the QuickDASH score. Secondary outcomes were the numerical rating scale (NRS) pain score, complications, and implant removal.

**Results:**

A total of 67 patients (88%) were available for the final follow-up. There was a significant difference in bicortical lateral fragment size, 15 mm (± 4, range 6–21) in the CPH group compared to 20 mm (± 8, range 8–43) in the SCPLE group (*p* ≤ 0.001). There was no significant difference in median QuickDASH score (CHP; 0.00 [IQR 0.0–0.0], SCPLE; 0.00 [IQR 0.0–4.5]; *p* = 0.073) or other functional outcome scores (NRS at rest; *p* = 0.373, NRS during activity; *p* = 0.559). There was no significant difference in median QuickDASH score or other functional outcome scores between Neer type II and type V fractures. There was no significant difference in complication rate, CHP 11% and SCPLE 8% (relative risk 1.26; [95% CI 0.25–6.33; *p* = 0.777]). The implant removal rate was 100% in the CHP group compared to 42% in the SCPLE group (relative risk 2.40; [95% CI 1.72–3.35; *p* ≤ 0.001]).

**Conclusion:**

Both the CHP and SCPLE are effective fixation methods for the treatment of unstable LCF, resulting in excellent patient-reported functional outcome and similar complication rates. SCPLE fixation is an effective fixation method for the treatment of both Neer type II and type V LCF. The SCPLE has a lower implant removal rate. Therefore, if technically feasible, we recommend SCPLE fixation for the treatment of unstable LCF.

## Introduction

The fracture of the clavicle is frequently encountered in the emergency department, accounting for 2.6–4% of fractures in the adult population. Furthermore, clavicle fractures represent 35–44% of fractures in the shoulder region. Although the majority involve the midshaft, lateral fractures account for 10–30% [1–6].

Lateral clavicle fractures (LCF) are classified according to Neer based on their relation to the coracoclavicular ligaments [6, 7]. Neer types I, III and IV are considered to be stable fractures and are generally treated conservatively. The unstable Neer type II and V fractures account for approximately 10–52% of LCF. Surgical management is recommended for these unstable LCF, as non-operative treatment results in a 22–50% non-union rate [1–6, 8, 9].

Neer type II fractures are unstable due to the detachment of the coracoclavicular ligaments from the medial fragment. Neer type V fractures have a comminuted character, with only an inferior fragment remaining attached to the coracoclavicular ligament [4, 6, 7].

Fixation of LCF proves to be a challenge as it can be difficult to get a firm hold on small lateral fragments. In addition, opposing forces contribute to considerable displacement of the fracture ends. Therefore, LCF can usually only be stabilized by rigid fixation methods [4, 9]. Different surgical fixation methods are available for the treatment of unstable LCF. However, at present, no consensus has been reached regarding the optimal fixation method.

The clavicle hook plate (CHP) is fixated with a small hook under the acromion posterior to the acromioclavicular joint. Complications related to the CHP such as acromial osteolysis, acromion fractures, rotator cuff tears and sub-acromial impingement have been reported [4, 5, 10, 11].

The superior clavicle plate with lateral extension (SCPLE) is a more recently developed locking compression plate. The SCPLE has multiple locking screws on the lateral end, divergently configured to maximize screw purchase on LCF fragments. The SCPLE does not interfere with the acromioclavicular joint and has a relatively low-profile [12–17]. Previous case series have shown the SCPLE to be an effective fixation method for the treatment of unstable Neer type II fractures [12–17]. However, the results after SCPLE fixation of Neer type V fractures have not yet been studied.

Currently, both the CHP and SCPLE are being used for the treatment of LCF. However, definitive consensus and guidelines for the surgical fixation of LCF have not yet been established. The aim of this study was to retrospectively evaluate patients treated with CHP and SCPLE fixation by comparing patient-reported functional outcome, complication-, and implant removal rates. Our hypothesis was that the SCPLE would result in better functional outcome and would lead to a reduction in complication- and implant removal rates.

## Materials and methods

### Study design

A retrospective cohort study was performed using data from two level II trauma centers. All patients with an unstable LCF who were treated operatively between January 2011 and June 2016 were eligible for inclusion. Inclusion criteria were: (1) acute LCF, (2) age 18 years or older, (3) Neer type II or type V fracture, (4) fixation with CHP or SCPLE, (5) fixation within 2 weeks of injury, and (6) minimum of one-year follow-up. Exclusion criteria were: (1) history of prior shoulder injuries or (2) neurovascular disorders of the affected shoulder. Data collection was performed by reviewing electronic medical records, operative reports, radiology reports, and telephone interviews by an independent research fellow. Electronic medical records were reviewed to collect baseline characteristics regarding affected shoulder, age, gender, trauma date, trauma mechanism, time from injury to surgery, fixation method, previous shoulder injuries, and lateral fragment size. Lateral fragment size was measured in millimeters (mm) on the anterior–posterior view radiograph. Overall lateral fragment size was defined as the total length of the largest lateral fragment. The largest intact bicortical fragment, which would allow for adequate screw fixation, was considered as the bicortical lateral fragment length. Informed consent was obtained from all subjects, and approval was granted by the institutional review board.

### Surgical procedure

Patients were treated by means of open reduction and internal fixation (ORIF) using a CHP (3.5 mm LCP; Depuy Synthes GmbH, Oberdorf, Switzerland) or SCPLE (3.5/2.7 mm LCP; Depuy Synthes GmbH, Oberdorf, Switzerland). Implant selection was based on the surgeon’s preference. CHP and SCPLE fixation were performed by several surgeons in both trauma centers. Operations were performed under general anesthesia with the patient placed in a beach chair position. An incision was made using a standard superior approach. The fracture site was exposed preserving as much periosteum as possible. Reduction was performed under direct visualization, and fragments were temporarily fixated using K-wires or reduction forceps. Fracture reduction, implant position, and screw placement were checked under fluoroscopic guidance. Coracoclavicular ligament repair was not routinely performed. Finally, the fascia and skin were closed in layers.

### Clavicle hook plate

**Fig. 1:**
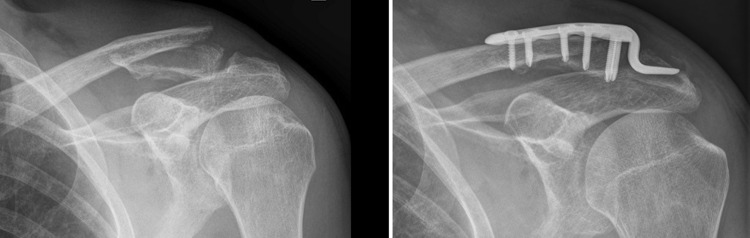
Preoperative radiograph of LCF and postoperative radiograph after CHP fixation

In cases of CHP fixation, a small incision was made in the posterior capsule of the acromioclavicular joint to allow sub-acromial hook placement. Trial plates were used to determine correct length and depth. Definitive CHP fixation was completed with the insertion of 3.5 mm angular stable or conventional screws (Fig. 1).

### Superior clavicle plate with lateral extension

**Fig. 2:**
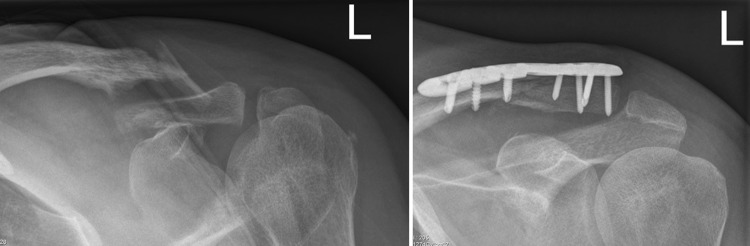
Preoperative radiograph of LCF and postoperative radiograph after SCPLE fixation

In cases of SCPLE fixation, there was no involvement of the acromioclavicular joint. A plate with an appropriate length was chosen to allow adequate fixation with 3.5 mm conventional or angular stable screws in the medial fragment and smaller 2.7 mm angular stable screws in the lateral end (Fig. 2).

### Postoperative management

Both groups received the same postoperative management. Radiographs were taken 1 day postoperatively. Patients were temporarily immobilized in a sling until the pain subsided; early mobilization and active range of motion exercises were allowed when tolerated. Weight-bearing activities and resisted exercises were not permitted until approval from the treating surgeon. Follow-up visits were scheduled at 2, 4, and 12 weeks postoperatively. Additional outpatient visits were scheduled depending on fracture consolidation. Removal of the SCPLE was not routinely performed, as opposed to the CHP where removal was recommended to all patients.

### Primary outcome

Functional outcome was assessed at least 12 months following ORIF, using the Dutch language version of the QuickDASH score. The QuickDASH is a validated and shortened version of the Disabilities of the Arm, Shoulder and Hand questionnaire (DASH). The QuickDASH is a patient-reported outcome instrument developed to measure upper extremity disability and symptoms, resulting in a score ranging from 0 (no disability) to 100 (most severe disability) [18, 19].

### Secondary outcome

Secondary outcomes were the numerical rating scale (NRS) pain score at rest and during activity, complications, revision surgery and implant removal. The NRS is a reliable and commonly used 11-point scale to measure pain intensity, ranging from 0 (no pain) to 10 (worst imaginable pain) [20]. Complications included infection, non-union, mal-union, implant failure, and implant removal-related complications. Infections were subdivided in superficial-skin or deep-wound infection. Superficial infection was defined as redness, swelling, or purulent discharge from the wound that was treated with antibiotics alone. If surgical irrigation and debridement was required, it was considered a deep infection. Non-union was defined as the absence of fracture consolidation 6 months after surgery. Mal-union was defined as a symptomatic deformity of the clavicle. Implant failure was defined as implant displacement, implant breakage, or breakage of screws. Revision surgery was defined as the need for subsequent surgery other than implant removal. Infection and re-fracture following implant removal were considered implant removal-related complications. Implant-related irritation and indication for implant removal were analyzed using a series of questions developed by Hulsmans et al. [21]. Responses to these questions allowed categorization of implant removal into (1) routinely or on patient’s request without irritation or (2) patient’s request due to irritation. Patients with the implant still in situ received a different series of questions, leading to categorization of why implant was not removed; (1) not experiencing irritation, (2) experiencing irritation but removal not necessary, (3) experiencing irritation but no request for removal due to fear of re-operation, or (4) experiencing irritation, considering removal.

### Statistical analysis

Descriptive results are presented as mean values with standard deviations and range (± SD, range) and median values with interquartile range (IQR) or absolute numbers and percentages (%). Continuous variables were evaluated using an independent sample *t* test or Mann–Whitney *U* test. Categorical variables were compared using the Pearson’s Chi-squared test. The Fisher’s exact test was used in case of small count sizes. Mean differences and relative risks (RR) were calculated with 95% confidence intervals (CIs). The significance level was defined as a *p* value < 0.05. All statistical analyses were performed using IBM SPSS Statistics version 24 for Windows (IBM Corp, Armonk, NY).

## Results

### Study population

**Fig. 3:**
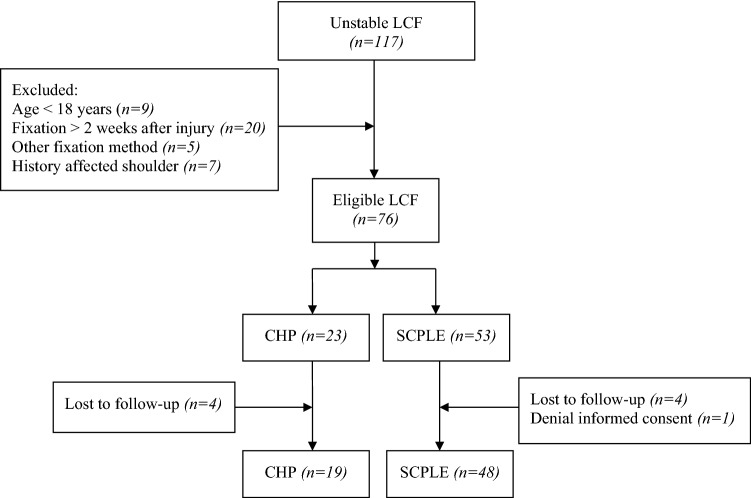
Flowchart representing patient selection for analysis of CHP versus SCPLE for unstable LCF

A flowchart of the patient cohort is shown in Fig. 3. In total, 76 patients met the inclusion criteria. However, eight patients could not be contacted, and one patient refused participation. This resulted in the inclusion of 67 patients (88%) for analysis. The baseline characteristics are shown in Table 1. The CHP group included 19 patients (28%) compared to 48 patients (72%) in the SCPLE group. The most frequent fracture pattern was Neer type II found in 43 patients (64%). The overall lateral fragment size was 39 mm (± 12, range 14–83). There was a significant difference in bicortical lateral fragment size, 15 mm (± 4, range 6–21) in the CPH group compared to 20 mm (± 8, range 8–43) in the SCPLE group (*p* ≤ 0.001). The mean time from injury to surgery was 6.9 days (± 3.6, range 0–14). The mean follow-up was 37.5 months (± 17.9, range 12–76).

**Table 1:** Baseline characteristics

	Overall *n* (%)^a^	CHP *n* (%)^a^	SCPLE *n* (%)^a^	95% CI of the difference	*p* value
Patients	67	19	48		
Age [mean ± SD]	43 (14)	42 (17)	43 (12)	−8.29 to 6.54	0.814
Gender					
Male	54 (81)	13 (68)	41 (85)		0.169
Female	13 (19)	6 (32)	7 (15)		
Side injury					
Left	39 (58)	8 (42)	31 (65)		0.108
Right	28 (42)	11 (58)	17 (35)		
Affected side dominant side					
Yes	27 (40)	10 (53)	17 (35)		0.270
No	40 (60)	9 (47)	31 (65)		
Neer classification					
Type II	43 (64)	13 (68)	30 (63)		0.780
Type V	24 (36)	6 (32)	18 (38)		
Overall lateral fragment (mm) [mean ± SD]	39 (12)	37 (12)	40 (12)	− 9.39 to 3.55	0.371
Bicortical lateral fragment (mm) [mean ± SD]	19 (7)	15 (4)	20 (8)	− 8.40 to 2.64	**< 0.001**
Time injury to surgery (days) [mean ± SD]	6.9 (3.6)	7.5 (3.5)	6.7 (3.6)	− 1.15 to 2.72	0.419
Follow-up (months) [mean ± SD]	37.5 (17.9)	31.3 (16.3)	40.0 (18.0)	− 18.25 to 0.77	0.071

^a^Percentages may not add up to 100 due to rounding. Bold values indicate statistically significant results (e.g., *p* < 0.05)

### Functional outcome

There was no significant difference in functional outcome, as shown in Table 2. The median QuickDASH score in the CHP group was 0.00 (IQR; 0.0–0.0), as opposed to 0.00 (IQR; 0.0–4.5) in the SCPLE group (*p* = 0.073). There were 15 patients (79%) with a QuickDASH score of 0 in the CHP group (range 0–21) compared to 25 patients (52%) in the SCPLE group (range 0–23). The median NRS pain score at rest was 0.00 (IQR; 0.0–0.0) in the CHP group and 0.00 (IQR; 0.0–0.0) in the SCPLE group (*p* = 0.373). There were 16 patients (84%) with a NRS pain score at rest of 0 in the CHP group (range 0–6) compared to 44 patients (94%) in the SCPLE group (range 0–3). In the CHP group, the median NRS pain score during activity was 0.00 (IQR; 0.0–1.0) compared to 0.00 (IQR; 0.0–2.0) in the SCPLE group (*p* = 0.559). There were 14 patients (74%) with a NRS pain score during activity of 0 in the CHP group (range 0–8) compared to 30 patients (63%) in the SCPLE group (range 0–7).

**Table 2:** Functional outcome and implant-related complications

	CHP (*n* = 19) *n* (%)^a^	SCPLE (*n* = 48) *n* (%)^a^	Relative risk (95% CI)	*p* value
QuickDASH median [IQR]	0.00 (0.0–0.0)	0.00 (0.0–4.5)		0.073
QuickDASH distribution [range]	0–21	0–23		
0	15 (79)	25 (52)		
0–10	3 (16)	19 (40)		
10–20	0	3 (6)		
20–25	1 (5)	1 (2)		
NRS pain at rest [median, IQR]	0.00 (0.0–0.0)	0.00 (0.0–0.0)		0.373
NRS pain at rest distribution [range]	0–6	0–3		
0	16 (84)	44 (92)		
0–3	2 (11)	3 (6)		
3–6	1 (5)	1 (2)		
NRS pain during activity [median, IQR]	0.00 (0.0–1.0)	0.00 (0.0–2.0)		0.559
NRS pain during activity distribution [range]	0–8	0–7		
0	14 (74)	30 (63)		
0–3	1 (5)	7 (15)		
3–6	2 (11)	8 (17)		
6–8	2 (11)	3 (6)		
Complications	2 (11)	4 (8)	1.26 (0.25–6.33)	0.777
Complication classification				0.929
Implant failure	1 (5)	3 (6)		
Non-union	1 (5)	1 (2)		
Revision surgery	1 (5)	2 (5)	1.26 (0.12–13.13)	1.000

^a^Percentages may not add up to 100 due to rounding. QuickDASH score: 0 = no disability to 100 = most severe disability. NRS pain score: 0 = no pain to 10 = worst imaginable pain. Bold values indicate statistically significant results (e.g., *p* < 0.05)

### Functional outcome according to Neer type

In both treatment groups, there was no significant difference in median QuickDASH score or other functional outcome scores between the Neer type II and type V fractures (Table 3). The median QuickDASH score in the Neer type II group following CHP fixation was 0.00 (IQR; 0.0–2.3), as opposed to 0.00 (IQR; 0.0–0.6) in the Neer type V group (*p* = 0.623). In the SCPLE group, the median QuickDASH score in the Neer type II group was 0.00 (IQR; 0.0–5.1), as opposed to 2.30 (IQR; 0.0–4.5) in the Neer type V group (*p* = 0.764).

**Table 3:** Functional outcome according to Neer classification

Neer	Type II	Type V	*p* value
CHP n (%)^a^	13 (68)	6 (32)	
QuickDASH median [IQR]	0.00 (0.0–2.3)	0.00 (0.0–0.6)	0.623
NRS pain score at rest [median, IQR]	0.00 (0.0–0.0)	0.00 (0.0–0.0)	1.000
NRS pain score during activity [median, IQR]	0.00 (0.0–2.0)	0.00 (0.0–2.0)	0.734
SCPLE n (%)^a^	30 (63)	18 (38)	
QuickDASH median [IQR]	0.00 (0.0–5.1)	2.30 (0.0–4.5)	0.764
NRS pain score at rest [median, IQR]	0.00 (0.0–0.0)	0.00 (0.0–0.0)	0.609
NRS pain score during activity [median, IQR]	0.00 (0.0–3.3)	0.00 (0.0–1.0)	0.100

^a^Percentages may not add up to 100 due to rounding. QuickDASH score: 0 = no disability to 100 = most severe disability. NRS pain score: 0 = no pain to 10 = worst imaginable pain. Bold values indicate statistically significant results (e.g., *p* < 0.05)

### Implant removal

Implant removal rates and indications are presented in Table 4. CHP fixation was associated with a significant higher removal rate. CHP removal was, according to protocol, performed in all 19 patients (100%) compared to 20 patients (42%) in the SCPLE group (relative risk 2.40; 95% CI 1.72–3.35; *p* ≤ 0.001). The mean time to removal was 4.3 months (± 2.2, range 2–10) and 13.6 months (± 11.5, range 5–50) in the CHP and SCPLE groups, respectively (mean difference − 9.287; 95% CI − 14.757 to 3.817; *p* = 0.002). In the CHP group, three patients (16%) reported removal without irritation and 16 patients (84%) reported removal due to irritation. There were no cases of implant removal-related complications. In the SCPLE group, 28 patients (58%) did not have the implant removed and 12 patients (43%) reported not to experience irritation.

**Table 4:** Implant removal rate and indication

	CHP (*n* = 19) *n* (%)	SCPLE (*n* = 48) *n* (%)	Mean difference (95% CI)	Relative risk (95% CI)	*p* value
Implant removal	19 (100)	20 (42)		2.40 (1.72–3.35)	**< 0.001**
Reason implant removed					0.695
Routinely or patient’s request, without irritation	3 (16)	5 (25)		0.63 (0.17–2.29)	
Due to irritation	16 (84)	15 (75)		1.23 (0.81–1.55)	
Time to implant removal (months) [mean ± SD]	4.3 (2.2)	13.6 (11.5)	− 9.287 (− 14.757 to 3.817)		**0.002**
Status implant not removed					NP
Not experiencing irritation	0	12 (43)			
Irritation, but implant removal not necessary	0	6 (21)			
Irritation, no request removal due to fear re-operation	0	5 (18)			
Irritation, considering removal	0	5 (18)			

Bold values indicate statistically significant results (e.g., *p* < 0.05). NP statistical analyses are not possible because all CHP implants were removed

### Complications

Complications were reported in two patients (11%) in the CHP group compared to four patients (8%) in the SCPLE group (relative risk 1.26; 95% CI 0.25–6.33; *p* = 0.777) (Table 2). Complications in the CHP group consisted of one case of implant failure due to implant displacement and one case of non-union. Complications in the SCPLE group included three cases of implant failure and one case of non-union. The implant failures in SCPLE group consisted of two implant displacements and one case of screw breakage. No cases of infection or mal-union were observed. In total, there were three patients that needed revision surgery. In the CHP group, one patient received a lateral clavicle resection due to non-union. Two revision surgeries were performed in the SCPLE group, one due to severe implant displacement and one case of non-union. The SCPLE implant displacement was treated by repeat SCPLE fixation. The non-union was treated with temporary K-wires fixation for 9.5 months.

## Discussion

There was no significant difference in patient-reported functional outcome or complication rate between CHP and SCPLE fixation. However, the CHP was used more often on fractures with a small lateral bicortical fragment. There was no significant difference in patient-reported functional outcome between Neer type II and type V LCF fractures. Furthermore, there was a significant higher implant removal rate in the CHP group. In the SCPLE group, 57% of patients with the implant still in situ reported varying degrees of implant-related irritation.

Both the SCPLE and CHP result in excellent functional outcome. These findings are in accordance with previous comparative studies. Zhang et al. [22] compared functional outcome of 36 patients with the SCPLE implant to 30 patients with the CHP using the Constant–Murley score and demonstrated no significant difference between groups. Erdle et al. [23] compared the results of 19 patients with CHP and 13 patients with SCPLE fixation, and they reported no significant difference between the groups when using the Constant score, the Oxford shoulder score, and the subjective shoulder value.

In the current study, the bicortical lateral fragment size was significantly smaller in the CHP group. Erdle et al. [23] reported no significant difference in lateral fragment size; however, they did not report whether the intact lateral fragment was bicortical. In the current study, the largest intact bicortical lateral fragment size which would allow for adequate screw fixation was measured. Our results indicate implant selection was influenced by the bicortical lateral fragment size. We recommend further research to focus on lateral fragment size to determine whether lateral fragment size negatively affects functional outcome and complication rates with the use of different implants.

Previous case series have shown the SCPLE to be an effective fixation method for the treatment of unstable Neer type II fractures [12–17]. Zhang et al. [22] treated fractures with a lateral fragment size larger than 2 cm with the SCPLE, and comminuted fractures close to the acromioclavicular joint were treated with the CHP with additional ligament repair. The comparative study by Erdle et al. [23] only included Neer type IIb fractures. To our knowledge this is the first study to evaluate the use of SCPLE fixation for the treatment of Neer type V fractures. In the current study, treatment with SCPLE fixation resulted in good functional outcome in both 30 patients (63%) with Neer type II and 18 patients (38%) with Neer type V fractures. These findings indicate SCPLE fixation is also an acceptable treatment option for acute Neer type V fractures, despite their comminuted character.

There was no a significant difference in complication rate between CHP and SCPLE fixation, which is in contrast to previous comparative studies. Zhang et al. [22] found a significantly higher complication rate, 23.3% in the CHP group compared to 5.6% in the SCPLE group (*p* = 0.04). However, Zhang et al. [22] included symptomatic hardware as a complication, and they reported three cases (10%) of symptomatic hardware in the CHP group and none in the SCPLE group. Erdle et al. [23] also reported a significantly higher overall prevalence of complications in the CHP cohort (89%) compared to the SCPLE cohort (38%) (*p* = 0.014). Erdle et al. [23] included radiographical proof of persistent acromial osteolysis and posttraumatic acromioclavicular joint arthrosis as complications.

The previous comparative studies included complications such as acromial osteolysis, posttraumatic acromioclavicular joint arthrosis, and sub-acromial impingement syndrome. These complications could be regarded as CHP implant specific. The CHP is fixated with a small hook under the acromion, posterior to the acromioclavicular joint which acts as a lever and maintains fracture reduction. However, this mechanism not only limits abduction of the arm, it may also affect the acromion and induce discomfort. The SCPLE does not interfere with the acromioclavicular joint, which results in the absence of acromial and impingement complications. Furthermore, there are several reports that indicated that these CHP implant-specific complications can resolve after removal [11, 24]. Renger et al. [11] evaluated the use of the CHP in 44 patients, and 30 patients (68%) reported implant-related discomfort. Renger et al. [11] found all implant-related complaints and osteolytic defects to disappear after implant removal.

Implant-related irritation and implant removal were analyzed using the series of questions developed by Hulsmans et al. [21]. In the current study, all CHP implants were removed after a mean of 4.3 months, in line with previous studies recommending CHP removal after fracture consolidation [11]. The comparative study by Zhang et al. [22] reported all CHPs were removed compared to 12 SCPLEs (33%). Erdle et al. [23] reported CHP removal was recommended and all CHP implants were removed after a mean period of 4.7 months. In the Erdle et al. [23] study, 77% of SCPLE implants were removed after a mean period of 12.5 months due to local irritation or on patient’s explicit request. In the current study, after a minimum of 12 months following ORIF, 42% of SCPLE implants were removed. Moreover, 43% of the patients with the SCPLE still in situ reported not to experience any irritation.

This study has some limitations. First, the study is limited by the retrospective nature. This study did not include prospective collection of functional and radiological measures during different follow-up times, which would increase the understanding of the impact implants have prior to implant removal. Second, fixation method was based on surgeon’s preference, which could cause bias through selection-by-indication. Therefore, different measurements were performed to determine whether lateral fragment size influenced implant selection. Finally, our study is limited by the small number of included patients in the treatment groups. However, this number is in accordance with previous comparative studies. Unfortunately, results after the use of CHP and SCPLE fixation have not yet been widely studied.

To our knowledge, this is the first study to evaluate the use of the CHP and SCPLE, focusing solely on implant selection without major differences in surgical technique or ligament repair. Furthermore, this is the first study to present the results of SCPLE fixation for the treatment of both Neer type II and type V fractures. Unfortunately, comparison of literature remains difficult due to small sample sizes, wide variety of functional outcome scores, definitions and surgical techniques. Therefore, a large multicenter study might provide insight into long-term results following different treatment modalities, influence of different LCF fractures types, and different patient populations.

## Conclusion

Both the CHP and SCPLE are effective fixation methods for the treatment of unstable LCF resulting in excellent patient-reported functional outcome and similar complication rates. SCPLE fixation is an effective surgical fixation method for the treatment of both Neer type II and type V LCF. The SCPLE has a lower implant removal rate compared to the CHP. Therefore, if technically feasible, we recommend SCPLE fixation for the treatment of unstable LCF.
